# Influence Factors on the Hepatotoxicity of *Polygoni Multiflori Radix*

**DOI:** 10.1155/2019/5482896

**Published:** 2019-09-25

**Authors:** Yun Wei, Meixia Liu, Jiangang Liu, Hao Li

**Affiliations:** Xiyuan Hospital of China Academy of Chinese Medical Sciences, Beijing 100091, China

## Abstract

**Background:**

Chinese herbal medicine (CHM) with reported hepatotoxicity is identified, in which *Polygoni Multiflori Radix* (HSW) attracts most attention. According to the Traditional Chinese Medicine (TCM) theory, processing is believed to be able to reduce the toxicity of HSW, but in publications, both processed and unprocessed HSW are reported to cause liver injury.

**Methods:**

This article reviews the case reports and experimental researches involving liver damage associated with HSW from the following aspects: clinical features, hepatic toxicity components, hepatotoxicity mechanism, and so on.

**Results:**

HSW has hepatotoxicity in different degrees and even leads to death, and the reason is multioriginal.

**Conclusions:**

People should be educated to have a broad understanding on ensuring drug use safety and lower drug-induced risks when taking HSW preparations.

*Polygoni Multiflori Radix* (He Shou Wu in Chinese pinyin; derived from the tuberous roots of *Polygonum multiflorum* Thunb., hereinafter referred to as HSW), as a traditional Chinese herb, is an example of Traditional Chinese Medicine (TCM) used over centuries for the treatment of various kinds of systemic diseases in China. HSW has been acclaimed for various biological activities, including antioxidation, radical scavenging activity, nerve cell protection, lipid regulation [1], and hair-follicle growth [2]. But **i**n recent years, the clinical efficacy of bioactive products, especially the safety of HSW and HSW-containing herbal products, has received much attention; due to the increasing reports of various cases on the adverse effects of hepatotoxicity published worldwide, reports on hepatotoxicity covered raw HSW (RHSW), processed HSW (PHSW), and traditional Chinese medicine (TCM) preparations containing HSW and healthy food. But the mechanism of HSW-induced hepatotoxicity is still unclear.

## 1. Function

In the *Chinese Pharmacopoeia* (2010), there are two forms of HSW decoction pieces: raw state (natural root, RHSW for short) and processed form, that is, processed HSW (boiled in black-bean liquid according to a traditional process, PHSW for short). During processing, significant changes in chemical profiles occur, which inevitably influence the associated pharmacological properties of HSW. The two forms have different properties based on the theory of TCM: RHSW is used for detoxification, eliminating carbuncle, preventing malaria, and loosening the bowel, whereas PHSW is used for tonifying the kidney and liver, supplementing essence and blood, blackening hair, strengthening bones and muscles, eliminating dampness, and reducing lipid [3]. In addition, the stem and leaf of HSW can be also used as medicinal material. In 2002, RHSW and PHSW have been authorized as healthy food additives by the government of the People's Republic of China.

Although HSW is usually used to treat various liver diseases, an increasing number of cases of potential HSW-induced hepatotoxicity resulting in severe drug-induced liver injury and even death have been reported. As a Chinese herbal medicine, HSW is a multicomponent synergistic system, which might play both therapeutic and toxic roles in humans. How HSW produces these biphasic effects (liver injury and liver protection) remains to be determined.

## 2. Diagnosis

The study of herb-induced liver injury (HILI) is difficult and controversial. An increasing number of HILI cases have been reported in recent years, presenting new clinical challenges. The accepted cause of HILI is chemical compounds, which are either produced by the plant, synthetic processes, or drug interaction. Whether risk factors such as high lipophilicity, genetic background of patients, or high daily and cumulative doses play a pathogenetic role for HILI is uncertain. HILI is not restricted to Chinese herbs but may be commonly observed in virtually all countries where herbs are consumed.

HILI is a diagnosis of exclusion because clinical features of HILI are lack of reliability of serologic or histological tests, compared with liver injury induced by virus, alcohol, and other causes [4]. HILI can present the same clinical symptoms and pictures of liver injury as drug-induced liver injury (DILI). So the diagnosis of HILI is established on the basis of drug history before the symptomatic period, physical examination on admission, and acute hepatitis according to laboratory investigations or histological findings.

HSW is widely used in China and other countries now for different clinical applications. Hundreds of liver injury cases related to HSW have been reported from all over the world, so the drug regulatory department in our country pays unprecedented attention, and the National Library of Medicine's LiverTox database of the United States also sets up the column of HSW-induced liver injury.

## 3. Clinical Characteristics

Several cases of HILI caused by the administration of HSW have been reported, particularly in mainland China, Hong Kong, Korea, Japan, Canada, Britain, and Australia. Clinical symptoms of HSW hepatotoxicity in traditional and modern medicine are variable, but epidemiology research studies have found that patients with liver injury caused by HSW more common are female and the history of drinking alcohol is rare, so the hepatocellular symptoms are the main type of liver injury symptoms caused by HSW [5]. Because symptoms are mostly unspecific and sometimes difficult to direct to the liver, early recognition of the unfolding liver injury is delayed. It can be seen that HSW and its preparations may cause damage to the liver and bile system, gastrointestinal system, skin and respiratory system, and other systems. In detail, the most common symptom is fatigue, besides, dark urine, nausea, and abdominal pain are also numerous, but signs such as rash, pruritus, mild eosinophilia, and clay-colored stools have also been reported [6] (see Table 1). Clinical signs may emerge alone or in combination with other features, while jaundice is the symptom initially best recognized by the patients [7]. Most HSW-induced liver injuries are reversible after withdrawal of HSW products and corresponding treatments. However, it can also lead to liver failure, cirrhosis, and even death.

## 4. Pathogenesis of Liver Injury Caused by HSW

BiNGO analysis [29] has revealed that the HILI was closely related to the following four processes: apoptosis, the regulation of cell death and proliferation, the regulation of metabolic processes, and the regulation of signal transduction. HSW can influence the metabolism of toxic substances by disturbing the balance between these processes.

### 4.1. Polymorphism of Liver Metabolic Enzymes

Both, natural and synthetic chemicals are foreign products to the body and need metabolic degradation to be eliminated. The hepatotoxicity of HSW is closely related to metabolism. The liver is an important organ for drug metabolism, and drugs including HSW are mainly excreted through the drug metabolizing enzyme of liver. HSW can induce the metabolic disorders of energy metabolism and amino acid and lipid metabolism. All of these metabolic disorders consequently contribute to liver cell death. Studies [30] also have shown that xenobiotic metabolism by CYP450, arginine and proline metabolism, tryptophan metabolism, retinol metabolism, and linoleic acid metabolism have similar effects on human liver cell metabolism and cause hepatotoxicity. By inhibiting the mRNA expression of 5 subtypes of P450 enzyme, the enzyme activity of PHSW may be reduced, which may lead to the accumulation of some hepatotoxic components in the liver, resulting in different degrees of damage to the liver tissue. RHSW group of liver microsomal protein is significantly decreased and CYP450 content is increased significantly, while PHSW group has no obvious change [31].

### 4.2. Apoptosis

Based on a comprehensive analysis of existing clinical hepatotoxicity cases and literature reports, hepatotoxicity induced by HSW may be related to apoptosis caused by lipid peroxidation, immune injury, and cholestasis injury . However, the mechanism is not clear until now. One research has found that [32] the ethyl acetate extract and dichloromethane extract of HSW had significantly influenced the HepG2 cell number, nucleus area, mitochondrial mass, and mitochondrial membrane potential at the concentration of 1000 *μ*g/ml. Other research [33] also found that RHSW and PHSW could reduce the mitochondrial membrane potential and elevate levels of reactive oxygen species, glutathione, activating transcription factor 4, and superoxide dismutase 2. These researches have indicated that the hepatotoxicity of HSW may be related to mitochondria-mediated apoptosis according to the influence of HSW components on mitochondrial mass and the mitochondrial membrane potential.

### 4.3. Idiosyncratic Reactions

Some studies have suggested that the hepatotoxicity of HSW is probably idiosyncratic in patients, because the HSW-induced liver injury has the characteristics of sporadic injury, and there is no obvious correspondence between age, gender, dosage, time, and other factors. Immune-mediated liver injury can be seen as an immune response. The idiosyncratic hepatotoxic material and mechanisms attributed to HILI of HSW remain unknown. Some researchers have found that the ethyl acetate (EA) extract contained mostly trace amounts of anthraquinone glycoside (emodin-8-*O*-glucoside) and stilbenes, the chloroform (CH) extract contained only anthraquinones (mostly emodin-8-*O*-glucoside and emodin), and the residue (RE) extract contained neither anthraquinones nor stilbenes. The EA extract, but not the CH or RE extract, probably plays the major role in the pathogenesis of the idiosyncratic hepatotoxicity of HSW. Meanwhile, Li C and his team [34] reported that *cis*-THSG was closely associated with the idiosyncratic hepatotoxicity of HSW. Idiosyncratic hepatotoxicity of HSW is determined by the multicomponent and multitarget effect of HSW. We can discover immune enhancer and organism immune factors in HSW through figuring out the mechanism of idiosyncratic hepatotoxicity of HSW.

## 5. Biomarkers of Liver Injury Induced by HSW

In general [35], assessment for HSW-induced liver injury relies primarily on histopathology and clinical chemistry based on a panel of serum biomarkers, including aspartate aminotransferase (AST), alanine aminotransferase (ALT), alkaline phosphatase(ALP), *γ*-glutamyltransferase (GGT), and total bilirubin (TBil). While direct bilirubin (DBIL) and total bilirubin (TBIL) are more sensitive than ALT, AST, and ALP to liver injury induced by HSW [36]. However, histopathological diagnosis or serum biomarker mentioned above lacks sufficient sensitivity. So it is an urgent need to find new biomarkers for the diagnosis of HSW-induced liver injury.

Bile acids (BAs), derived from the catabolism of cholesterol, aid in breaking down fats and absorbing them into the body. A recent study [37] has found that the perturbation of nine BAs, which are generated in the liver and accumulated in blood during liver injury, is associated with HSW-induced liver injury; especially glycodeoxycholic acid (GDCA) in bile as well as hyodeoxycholic acid (HDCA) in serum can be selected as potential biomarkers for HSW-induced liver injury. But there are no reports about research on individual BAs in bile or serum in rats with liver injury induced by HSW. In another study [38], the researchers found that six potential biomarkers in rats with liver injury caused by HSW using the liquid chromatography-mass spectrometry method, including oleamide, lysoPC(16 : 0), leukotriene A4, trans-tetra-dec-2-enoic acid, dihydrocortisol, and 7a-hydroxydehydroepiandrosterone, were more sensitive than ALT and AST.

## 6. Possible Hepatic Toxicity Components

TCM often induces liver injury via its chemical components or metabolites. Therefore, it is imperative to identify hepatotoxicants and mechanisms of hepatotoxicity early and efficiently for the safe use of HSW. The main components in HSW are phenolic acids, diphenyl ethylene glycosides, flavonoids, and anthraquinones, of which 2,3,5,4′-tetrahydroxystilbene-2-*O*-*β*-*D*-glucopyranoside (THSG), emodin, and physcion are often used as maker compounds for quality evaluation. In addition, THSG, gallic acid, quercetin, kaempferol all exhibit antiaging effects and hepatoprotective activities in different pathological models. However, there is lack of research fully explaining which ingredient is the most important for liver toxicity.

Actually, compounds are isolated from HSW depending on different processing procedures. RHSW and PHSW possess different chemical constituents and pharmacological properties, which have been proved by modern pharmacological researches. Long processing time results in the chemical change of HSW, including the decreased contents of the emodin-8-*O*-(6′-*O*-malonyl)-glucoside, physcion-8-*O*-(6′-*O*-malonyl)-glucoside, THSG, emodin-8-*O*-*β*-*D*-glucopyranoside, and physcion-8-*O*-*β*-*D*-glucopyranoside [39] and the increased contents of emodin and physcion, meanwhile inducing the generation of hydroxymaltol, DDMP, and 5-HMF through Maillard reaction [40] (see Table 2). As shown in Wu team's work [41], the contents of characteristic compounds in RHSW are changed after processing: the content of THSG is decreased by 55.8%, whereas the content of emodin is increased by 34.0%. The reason is that stilbene glycosides and anthraquinones glycosides are unstable during processing, meanwhile the decocting influences the ratios of free anthraquinones and anthraquinones glycosides. So, a standardized processing procedure may be favorable for managing HSW use.

Many studies have also demonstrated that some quinones and stilbenes of HSW can lead to hepatotoxicity, especially emodin, physcion, and rhein [47]. Anthraquinones can form highly reactive anthrones in the colon, which results in hepatotoxicity. Other studies have found [48] that free anthraquinones (including emodin) and pinostilbenoside are not the main hepatocyte toxicity constituents of HSW due to their low content, but tetrahydroxystilbene-*O*-(galloyl)-hex and emodin-*O*-hex-sulfate. The toxicity of these two components requires further study, and the hepatotoxicity may result from a combination of active compounds rather than a single chemical entity (see Table 3).

## 7. Factors Contributing to HSW-Induced Liver Injury

### 7.1. Preparation Technique

Why there are so many reports on toxicology of HSW now compared with the ancient literature? There is rarely reported HSW toxicity in ancient herbal works despite the long application history in China. Different processing procedures used by manufacturers can lead to differences in the chemical constituents of PHSW. That may explain why changes in efficacy occur before and after processing. Most HSW products are unqualified according to the requirement of the Chinese Pharmacopeia which may take the responsibility for the toxicity of HSW [59]. Proper pharmaceutical processing may reduce toxicity or side effects and potentiate the beneficial effects through changing the pharmacological properties, preserving active constituents.

#### 7.1.1. The Time of Processing

As regards the processing time [44], the pharmacopoeia does not make clear provisions. Due to the limitations of compound species selection and the differences in determination methods, the appropriate processing time of HSW varies in different literatures. Studies have shown that [60] the contents of D-fructose, sucrose, and emodin in serum are decreased, and the content of D-glucose is increased with the extension of processing time. 4-5 h is the best processing time by high-performance liquid chromatography (HPLC). Another study [61] shows that 8～10 h is the best processing time for HSW with black bean juice stewing, because most active ingredients peak at this point. For instance, the content of THSG decreases with processing time and reached 17% of original at 48 h. The contents of tannin and combined and free anthraquinones also decrease with processing time.

#### 7.1.2. Decocting Times

The differences between ancient and modern processing methods are the potential cause of the increased events of HSW-induced liver damages. The ancient processing methods of HSW emphasize nine times of steaming and nine times of drying, while the modern processes have been simplified into one time of steaming. The research has found [46] that the content of trans-THSG gradually decreased after each processing cycle and is lowest after nine cycles, while the content of cis-THSG initially increased and only began to decrease after the fifth processing cycle to reach its lowest content after nine cycles.

#### 7.1.3. Varieties of Pharmaceutical Excipients

In the processing, a certain amount of supplementary materials can be used to mitigate or improve the efficacy of the drug. In the Chinese pharmacopoeia, the processing methods of HSW are steaming with black bean juice, braising with black bean juice, or steaming. Black bean can not only enhance the tonic effect of HSW but also significantly reduce its LD_50_. But other studies have found [62] that there is no marked difference for variously processed products in the main active components, anthraquinone and stilbene glucoside in PHSW.

#### 7.1.4. Different Solution Decoction

There is certain correlation between different solution decoction and the levels of liver injury of HSW. Several mechanisms, such as hydrolysis, dehydration, isomerization, and Maillard reaction appear to be involved in the preparation method of HSW. Both ethanol extracts and water extracts of HSW can induce significant lipid accumulation in Sk-Hep1 cells in a dose-dependent manner via reducing *ß*-oxidation and increasing de novo lipogenesis [49]. Another study [52] has showed that liver injury due to alcohol extraction is more serious than water extraction of HSW, and the toxicity level is probably 70% alcohol > 95% alcohol > 50% alcohol > 30% alcohol > water [63]. Clinically, water decoction is the major application form of HSW.

But the mechanism for liver injury caused by ethanol extracts and water extracts of HSW is different. Through gas chromatography-mass spectrometry (GC-MS), Zhang et al. [52] have found that the concentrations of myo-inositol, oleic acid, and cholesterol in water extraction groups increased, while those of hexadecanoic acid, octadecanoic acid, ribitol, and butanedioic acid decreased to a great extent. The concentrations of myo-inositol, phosphoric acid, uridine, oleic acid, cholesterol, and butanoic acid in alcohol extraction groups increased to a great extent, while those of hexadecanoic acid, octadecanoic acid, ribitol, and butanedioic acid decreased. Because of these results, RHSW and PHSW have different functions.

### 7.2. The Irrationality of Using HSW

#### 7.2.1. Dosages of HSW

As a typical tonic medicine, the clinical utility of HSW is largely limited by its standardization and reliability of processing methods in ancient Chinese medicine. The hepatotoxicity of HSW is not easily detected when used at recommended doses. In the Chinese Pharmacopoeia (2010 edition), the predetermined daily dose of RHSW is 3–6 g and PHSW is 6–12 g. The content limit for THSG in HSW is set lower in current version of pharmacopoeia. It is suggested that the content of THSG should be set no less than 2.0%, and emodin, emodin monoglucoside, physcione, and physcione-monoglucoside are no less than 0.15% in RHSW. In PHSW, THSG content should be set no less than 1.2%, while the anthraquinone content limit needs further research.

Clinical and animal experiments have proved that HSW associated with liver damage has a “dose-time-toxicity” relationship [64]. One work showed [54] that oral administration of the aqueous extract of HSW did not affect rat serum ALT or AST levels significantly even at a dosage of 40 g/kg body weight (BW) that is 400-fold the clinical dosage according to the current Chinese Pharmacopoeia. When PHSW is used at the dosage of 22 g/kg/day which is 10 times the normal intake for adult per day [65], there are no toxic or side effects.

#### 7.2.2. The Time of Usage

With continuous administration for 4∼8 weeks, HSW at higher doses is a major cause of acute liver failure and transplantation [66]. Patients with medication time ≥30 days have significantly higher positive rate of autoantibodies than those with medication time <30 days [67]. From above, it is recommended that RHSW should be taken continuously for no more than 2 weeks, and PHSW should be taken continuously for no more than 4 weeks. That is why HSW should not be used as long-term health care products.

#### 7.2.3. Cultivation and Harvesting of HSW

Cultivation and harvesting may also be one of the factors affecting the safety of HSW. The harvesting seasons of HSW vary from successive generations. Before the Song dynasty, it was mainly harvested in spring and summer, while after Qing dynasty, it was mainly harvested in winter. The rules of the Chinese pharmacopoeia on the harvesting of HSW (harvesting when leaves wither in autumn and winter) are the same as those in the Song dynasty. This conclusion has been confirmed by Shuaifeng Li' research team [68], and they have found that the contents of THSG in the HSW of different harvest seasons are as follows in descending order: September > October > August > November > December > January; the contents of conjugated anthraquinones reached the peak on September and then descended over time, while the contents of uridine, adenine, guanosine, and cytidine were higher in December.

#### 7.2.4. Herb-Drug Interactions

Herb-drug interactions are of great concern when patients concomitantly take drugs and herbs, especially taking herbal medicines and western medicines at the same time. A recent study [69] has found that THSG (200, 400, 800 mg/kg) exacerbated the hepatotoxicity induced by sub-toxic dose of acetaminophen (APAP) (200 mg/kg) in mice, but THSG alone had no hepatotoxicity. Its mechanism may be related to THSG by which the expression of CYP2E1, CYP3A4, and CYP1A2 is increased both in mice and in human normal liver L-02 hepatocytes. In another [70], the combined use of poria and HSW in the ratio of 1 : 2 can significantly ameliorate the HSW/lipopolysaccharide (LPS) -induced liver injury and systemic inflammation through arginine and proline metabolism, primary bile acid biosynthesis and sphingolipid metabolism, and inflammatory stress, whereas, radish seed can increase the liver injury of HSW. It is essential to study the feasibility of detoxification by compatibility of HSW.

### 7.3. Properties of HSW

#### 7.3.1. Locality of HSW

HSW is widely distributed in China's southwest, central, south, east, and other regions, including Sichuan, Yunnan, Guizhou, Chongqing, Guangdong, Guangxi, Jiangsu, Anhui, Hubei, Hunan, Henan, Jiangxi, Shanxi, Gansu, and other provinces and cities. So the distinction among HSW from different origins is essential for selecting good quality HSW to treat diseases. But owing to the increasing market demand in recent years, the wild resources of HSW are being seriously broken, which resulted in reducing the yield and quality of medicinal material. A recent study [48] has found that the toxicity of HSW, no matter RHSW or PHSW, is obviously different among the different geographical areas, and the most toxic HSW is from the Sichuan province when compared to Hunan, Guangdong, Shengzhou province. This phenomenon might indicate that the geography may be more suitable for HSW production.

#### 7.3.2. Specification of HSW

The specification features, including shape, size (diameter, length), color, and texture, may be linked with quality of HSW. One study indicates [71] that the contents of HSW vary in samples of different diameters and thicknesses. In contrast to those irregular thick slice samples of HSW, the contents of THSG, emodin, and physcion in the thin slices are low and sometimes undetectable. Among them, the hepatocyte toxicity of HSW powder is much greater than that of HSW lumps because of the higher solubility [72], especially pulverized powders, which therefore shall be given high attention. And HSW with broader cork and phloem [73], as seen in a transverse section, is typically of better quality as these parts are the situations of the bioactive component accumulation. This research also finds that stilbene glucosides, combined anthraquinones and polyphenols, are mainly distributed in the cork, and free anthraquinones are mainly distributed in the phloem by means of fluorescence microscopy and high-performance liquid chromatography time-of-flight mass spectrometry (HPLC-TOF-MS). In addition [74], there are close and direct correlations between the quantity of clusters of calcium oxalate in HSW and the contents of THSG and anthraquinone (CAQ), so HSW with a higher quantity of clusters of calcium oxalate should be of better quality.

Besides, different drying temperatures and drying methods have a greater impact on the content of effective components in HSW.

### 7.4. Human Factors

The routine dosage of HSW also can lead to liver injury, suggesting that the hepatic toxicity of HSW may not have a significant dose-toxicity relationship but may be related to atopic status and genetic background. Some experts [30] say that people with high enzymatic activity may be more susceptible to the potential adverse effects of HSW because the level of CYP1A2 significantly varied amongst the individuals, indicating that there is some inherited tendency to hepatocyte toxicity of HSW. Another new study finds that human leukocyte antigen (HLA)-B^*∗*^35 : 01 is probably a high-risk allele of HSW associated with liver injury [75]. These studies show that HSW is only at risk of liver injury for a small number of specific populations and is safe for the vast majority of populations, which lay a solid theoretical foundation for the scientific formulation of comprehensive prevention and control strategies for liver injury risk of HSW and its related preparations.

Investigations regarding HILI also suggest that women are more susceptible, especially for those of 60–65 years of age. The cause might be due to age- and sex-related differences in hepatic microsomal enzyme activity [6]. The counter-argument is that HSW associated with liver injury can occur at any age group and with no gender orientation. Besides, people with hepatobiliary diseases, diabetes, and hypertension are likely to increase drug susceptibility and more prone to cause liver injury [67]. In conclusion, the elderly and women with declining liver and kidney function and the children with weaker liver and kidney function should pay close attention to the process of taking HSW.

## 8. Conclusions and Future Perspectives

As one of the most widely used traditional medicines in East Asia and North America, HSW and its processed products have been widely used for the clinical treatment of fatty liver disease, hyperlipidemia, antiaging, cirrhosis, hepatitis B, learning and memory obstructions, and so on. Despite its long history, an increasing number of hepatotoxicity cases of HSW have been reported, presenting new clinical challenges. High doses of HSW have either an injurious effect on normal rats or a therapeutic effect on rats with chronic liver injury, indicating both harmful and protective effects on the liver. The problem is how to handle the balance of therapeutic and toxic effects on the liver of HSW. Toxicology studies on HSW are still limited, and further research is needed. But contradictory conclusions are drawn that might be caused by the inconsistent quality of market decoction pieces. To fully understand the characteristics and possible factors of hepatitis associated with HSW, we conduct a comprehensive review of the relevant published literatures.

As we know, the quality of herbal medicines can be affected by the mixture of controllable and uncontrollable factors, the former including geography, seasonal variation, cultivation, harvesting, storage, and postharvest treatment, processing procedure influence herb quality which further results in different pharmacological effects; human factors belong to the latter. As we discussed in this review, combined anthraquinones are unequivocally hydrolyzed into free ones, but the compounds from the degradation of THSG are difficult to detect. Knowing the detailed transformation of major compounds in HSW during the processing procedure could allow us to better understand the mechanisms of preparation, which would facilitate the establishment of a quality control method and the normalization of the processing technology. Therefore, the comprehensive chemical composition analysis, sample classification, and high-risk group are of great importance for reducing the hepatotoxicity of HSW. We hope that our findings can provide guidance for clinical medication and scientific research and thus can help to avoid hepatitis induced by HSW in the future.

## Figures and Tables

**Table 1:** Patient details recorded from published case reports from year 2000.

Author	Country	Duration of intake, day	Clinical manifestations	Outcome	HSW type
Park et al. [8]	Australia	14	Malaise, nausea, emesis, pruritus, dark urine, and jaundice	Recovery	Shou-Wu-Pian
Yuan [9]	China	7	Fatigue and aggravated jaundice	Recovery	Ointment made by RHSW
Mazzanti et al. [10]	Italy	30	Jaundice, nausea, abdominal pain, yellow skin, and dark urine	Recovery	Shou-Wu-Pian
Wong et al. [11]	Netherlands	120	Jaundice, dark urine, and pale stools	Recovery	Shou-Wu-Pian
Cárdenas et al. [12]	Colombia	56	Maculopapular rash and mild eosinophilia	Recovery	Shen-Min
Fu and Yan [13]	China	18	Without clinical symptoms	Recovery	Chinese herbal medicine soup containing PHSW
Laird et al. [14]	America	Several months	Nausea and tea-colored urine	Recovery	NuHair
Cho et al. [15]	Korea	30	Fatigue and aggravated jaundice and fever and progressive pancytopenia	Recovery	Tea with RHSW
Furukawa et al. [16]	England	240	Fatigue	Recovery	Shou-Wu-Pian
Valente et al. [17]	Italy	14	Right upper abdominal pain, fatigue, jaundice, and nausea	Recovery	Shou-Wu-Pian
Li and Zhang [18]	China	60	Jaundice and abdominal pain	Recovery	Drink water with RHSW
		75	Jaundice	Recovery	Drink water with RHSW
Chen et al. [19]	China	1hour	Nausea and tea-colored urine	Recovery	RHSW
Hu et al. [20]	China	30	Fatigue and tea-colored urine	Recovery	Shouwu Yanshou pian
Cortez et al. [21]	Asia	60–90	Malaise, diarrhoea, myalgias, and arthralgias	Expire	Ban Tu Wan
Banarova et al. [22]	Slovenská republika	60	Nausea and jaundice	Recovery	*Polygonum multiflorum* pills
Zhen and Zeng [23]	China	14	Fatigue, nausea, and jaundice	Recovery	He Shouwu mixtures
Miao and Yun [24]	China	20	Jaundice and tea-colored urine	Recovery	PHSW powder
		180	Fatigue and tea-colored urine	Recovery	Drink water with RHSW
Li et al. [25]	China	30	Nausea, low-grade fever and tea-colored urine, and jaundice	Recovery	Wine made by PHSW
Lei and Li [26]	China	60	Fatigue and jaundice	Recovery	Steamed white rice with HSW
Zhang et al. [27]	China	45	Jaundice	Recovery	RHSW powder infused with water
Li et al. [28]	China	30	Fatigue, poor appetite, dark urine, and jaundice	Recovery	Qibao Meiran Wan

**Table 2:** Different chemical constituents in RHSW and PHSW.

	Preparation method	Identified compounds	Country of origin	Conclusion	Observational method
Liang, et al. [39]	Softened by water and then steamed in an autoclave for four hours at 121°C and under 2.03 pounds per square inch	15	Guangdong and Hong Kong	The contents of emodin-8-*O*-(6′-*O*-malonyl)-glucoside and physcion-8-*O*-(6′-*O*-malonyl)-glucoside are disappeared or decreased significantly, and the contents of THSG, emodin-8-*O*-*β*-*D*-glucopyranoside and physcion-8-*O*-*β*-*D*-glucopyranoside are decreased after processing. On the other hand, the contents of emodin and physcion generally increased after processing.	HPLC and MS
Liu et al. [40]	Not mention	23	Not mention	The relative amounts of gallic acid, emodin, and physcion are very high in PHSW samples compared to those in RHSW samples; catechin, flavanol gallate dimer, polygonimitin B, emodin-1-*O*-glucoside, emodin-8-*O*-(6′-*O*-malonyl)-glucoside, and physcion-8-*O*-(6′-*O*-malonyl)-glucoside are disappeared after processing	RRLC/DAD/ESI-MS
Chen et al. [42]	Softened by water and then steamed, and dried at a low temperature	5	Deqing County, Guangdong province	The content of stilbene glycoside is decreased in the process of time during processing and both the dissociative anthraquinones, emodin and emodin monomethyl ether, are increased by processing; anthraquinone, emodin and emodin monomethyl ether, both of them are increased by processing, and after 32 h the content reaches the maximum.	RP-HPLC
Wang et al. [43]	Not mention	23	Different regions of China	The contents of sucrose, gallic acid, procyanidin B, catechin, THSG, torachrysone-*O*-glu, emodin-8-*O*-glu, and emodin-*O*-(malonyl)-glu are destroyed due to long-time steaming	UHPLC-LTQ-Orbitrap MS^n^
Zhao et al. [44]	Mixed with black bean juice and steamed by water	16	Sichuan	Compared with RHSW, the contents of procyanidine B1, catechin, epigallocatechin, Polydatin, Rhaponticin, resveratrol, rhein, THSG, emodin -8-*O*-*β*-*D*- glucoside, and physcion -8-*O*-*β*-*D*- glucoside are decreased after processing; the contents of gallate, emodin, and physcion are increased after processing. The content of other components remained unchanged.	HPLC
Yang et al. [45]	Nine cycles of steaming and sundrying	3	Hunan	The content of THSG decreased from 3.021% to 0.905%; the content of bombined anthraquinone decreased from 0.401% to 0.015%; the content of total anthraquinone decreased from 0.681% to 0.445%; the free anthraquinone content increased from 0.281% to 0.432%	HPLC
Liang et al. [46]	Nine cycles of steaming and sundrying	87	Kaili City in Guizhou	The contents of gallic acid continuously increased, while *trans*-THSG continuously decreased, after each processing cycle. The contents of emodin and physcion are first decreased and then increased with the increasing of processing cycles. In terms of the other eight analytes, namely *cis*-THSG, emodin-8-*O*-*β*-*D*-glucosides and physcion-8-*O*-*β*-*D*-glucosides, catechin, epicatechin, epicatechin-3-gallate, proanthocyanidin B1, and proanthocyanidin B2, the contents are first increased and then decreased after the increase of processing cycles.	UPLC-QTOF-MS/MS and UPLC-QTOF-MS/MS

HPLC: high-performance liquid chromatography; MS: mass spectrometry; RP-HPLC: reverse phase high-performance liquid chromatography; RRLC/DAD/ESI-MS: rapid resolution liquid chromatography-diode array detection/electrospray ionization tandem mass spectrometry; UPLC-QTOF-MS/MS: ultra-performance liquid chromatography-quadrupole/time-of-flight mass spectrometry; UPLC-QqQ-MS/MS: ultra-performance liquid chromatography-quadrupole/triple quadrupole mass spectrometry; HPLC–DAD–CL: high-performance liquid chromatography–diode array detection–chemiluminescence; UHPLC-LTQ-Orbitrap MS^n^: ultra-high-performance liquid chromatography coupled with electrospray ionization-linear ion trap-Orbitrap hybrid mass spectrometry.

**Table 3:** Possible hepatic toxicity components in HSW.

Author	Possible hepatic toxicity components	Conclusion
Furukawa et al. [16]	Anthraquinones and contaminants (mycotoxins, heavy metals, and pesticides)	The incorrect use of HSW might be the leading cause
Yu et al. [49]	Anthraquinones	The toxicity of water extractions of RHSW and PHSW < any other solvent (50% ethanol and 95% ethanol)
Wu et al. [41]	The content of tetrahydroxystilbene glucosides	The toxicity of water decocta > acetone extract. Meanwhile, the toxicity of acetone extract of RHSW > acetone extract of PHSW.
Lin et al. [47]	Anthraquinone, emodin-*O*-(malonyl)-hex, emodin-*O*-glc, emodin, emodin-8-*O*-glc, emodin-*O*-(acetyl)-hex, and emodin-*O*-hex-sulfate	RHSW ethanol extract > RHSW water extract > PHSW ethanol extract > PHSW water extract
Lv et al. [50]	Modin, physcion, EG, and physcion-8-*O*-*β*-*D*-glucoside (PG)	Ethanol extract has much stronger hepatotoxicity, the contents of emodin-8-*O*-*β*-d-glucopyranoside, physcion-8-*O*-*β*-d-glucopyranoside, emodin and physcion are significantly higher in ethanol extract than in water extract
Ma et al. [51]	Anthraquinones	Administration of high-dose HSW extract (20 g/kg) for 3 weeks can cause hepatic lesions, while the low-dose treatment (1 g/kg) is safe
Zhang et al. [52]	Vitamin A	The toxicity of alcohol extraction > water extraction.
Yang et al. [53]	Rhein, emodin, physcion-8-*O*-*β*-*D*-glucopyranoside, and physcion-8-*O*-(6′-*O*-acetyl)-*β*-*D*-glucopyranoside	Which anthraquinones are the prime constituents of the hepatotoxicity of HSW are still not be proved
Ma et al. [32]	Aloe emodin, emodin, rhein, and gallic acid	The anthraquinones of HSW have potential hepatotoxicity, and the liver injury caused by HSW is related to mitochondrial abnormalities.
Li et al. [54]	Emodin	HSW ethyl acetate extracts have close association with the idiosyncratic hepatotoxicity of HSW
Jiang et al. [55]	Anthraquinones	Conjugation with glutathione is a major detoxification route
Hong et al. [56]	Rhein	Caspase 3 (CASP3), peroxisome proliferator-activated receptor gamma (PPARG), and myeloid cell leukemia-1 (MCL1) are main potential targets for HSW-induced liver injury
Li et al. [34]	2,3,5,4′-tetrahydroxy cis -stilbene-2-*O*-*β*-glucoside (cis-THSG)	cis-THSG is closely associated with the idiosyncratic hepatotoxicity of HSW
Lin et al. [48]	tetrahydroxystilbene-*O*-(galloyl)-hex and emodin-*O*-hex-sulfate	The toxicity of the total fraction > the 30% ethanol fraction > the 70% ethanol fraction > the dichloromethane fraction and the water fraction
Wang et al. [30]	Some quinones and stilbenes, especially emodin and rhein	HILI may result from a combination of active compounds rather than a single chemical entity
Meng et al. [57]	*cis*-THSG combined with LPS	*cis*-THSG is the major factor responsible for HSW-induced liver injury, but it is not the only component
Quan, et al. [58]	Emodin, rhein, aloe emodin, emodin-1-*O*-glucoside, physcion-8-*O*-glucoside, and aloe emodin-8-*O*-glucoside	The hepatotoxicity of HSW may be mediated by anthraquinone compounds, and its material basis is related to the 6 anthraquinone compounds mentioned above, which are mainly on the combined anthraquinones

## Abbreviations

CHM: Chinese herbal medicine
HILI: Herb-induced liver injury
HSW: *Polygonum multiflorum* Thunb.
PHSW: Processed HSW
RHSW: Raw HSW
TCM: Traditional Chinese Medicine.
